# Preparation for Quarantine on the Cruise Ship Diamond Princess in Japan due to COVID-19

**DOI:** 10.2196/18821

**Published:** 2020-05-11

**Authors:** Yoshihiro Yamahata, Ayako Shibata

**Affiliations:** 1 Department of Emergency and Disaster Medicine Kyoto Prefectural University of Medicine Kyoto Japan; 2 Obstetrics & Gynecology Yodogawa Christian Hospital Osaka Japan

**Keywords:** SARS-CoV-2, COVID-19, infectious control, cruise ship quarantine, pandemic, outbreak, surveillance, preparation, infectious disease, public health, quarantine

## Abstract

**Background:**

Japan implemented a large-scale quarantine on the Diamond Princess cruise ship in an attempt to control the spread of the novel coronavirus severe acute respiratory syndrome coronavirus 2 (SARS-CoV-2) in February 2020.

**Objective:**

We aim to describe the medical activities initiated and difficulties in implementing quarantine on a cruise ship.

**Methods:**

Reverse transcription–polymerase chain reaction (RT-PCR) tests for SARS-CoV-2 were performed for all 3711 people (2666 passengers and 1045 crew) on board.

**Results:**

Of those tested, 696 (18.8%) tested positive for coronavirus disease (COVID-19), of which 410 (58.9%) were asymptomatic. We also confirmed that 54% of the asymptomatic patients with a positive RT-PCR result had lung opacities on chest computed tomography. There were many difficulties in implementing quarantine, such as creating a dividing traffic line between infectious and noninfectious passengers, finding hospitals and transportation providers willing to accept these patients, transporting individuals, language barriers, and supporting daily life. As of March 8, 2020, 31 patients (4.5% of patients with positive RT-PCR results) were hospitalized and required ventilator support or intensive care, and 7 patients (1.0% of patients with positive RT-PCR results) had died.

**Conclusions:**

There were several difficulties in implementing large-scale quarantine and obtaining medical support on the cruise ship. In the future, we need to prepare for patients’ transfer and the admitting hospitals when disembarking the passengers. We recommend treating the crew the same way as the passengers to control the infection. We must also draw a plan for the future, to protect travelers and passengers from emerging infectious diseases on cruise ships.

## Introduction

Since severe acute respiratory syndrome coronavirus 2 (SARS-CoV-2) was first detected in China on December 31, 2019, it has rapidly spread all over the world and 230,104 people have died from coronavirus disease (COVID-19) in 215 countries as of May 3, 2020 [1]. There have been many infections and related problems on ships worldwide such as the Grand Princess (United States) [2], the Ruby Princess and Ovation of the Seas (Australia), and Costa Luminosa (France). Japan implemented a large-scale quarantine on the Diamond Princess cruise ship, and all passengers including asymptomatic patients were tested for SARS-CoV-2 by using reverse transcription–polymerase chain reaction (RT-PCR); valuable lessons can be learned from the steps taken for quarantine implementation on the cruise ship. The Diamond Princess had 3711 people (2666 passengers and 1045 crew) on board, and the average age of passengers was 66.0 years [3]. The ship left Yokohama Port on January 20, 2020. A passenger who disembarked from the ship in Hong Kong on January 25 developed a fever on January 30. This passenger was confirmed to be positive for COVID-19 on February 1. The ship arrived at Yokohama earlier than scheduled on February 3, at which time the quarantine began [3].

We, the authors, worked as medical staff at the entrance of the Diamond Princess from February 14 to 17, 2020, when the number of RT-PCR–confirmed COVID-19 cases reached its peak. We supported the transport of people with positive RT-PCR results by coordinating with the hospital and transportation provider, depending on their condition. In addition to scheduling transportation, we arranged for emergency transportation of people whose symptoms worsened. Since February 18, Fujita Medical University Okazaki Medical Center (Shinkaiin Temple) has accepted a large number of patients with mild symptoms and has served as a place for quarantining asymptomatic patients.

We report on the experience of this large-scale quarantine and the passenger room isolation procedures implemented to control COVID-19 aboard a cruise ship. During the quarantine, RT-PCR testing of throat swabs was extended to all passengers in the following order:

1. Symptomatic patients and their close contacts
2. Elderly people aged 80 years or above and people with comorbidities
3. People aged 75 years and above
4. People aged 70 years and above
5. All other passengers
6. All crew members

**Figure 1:**
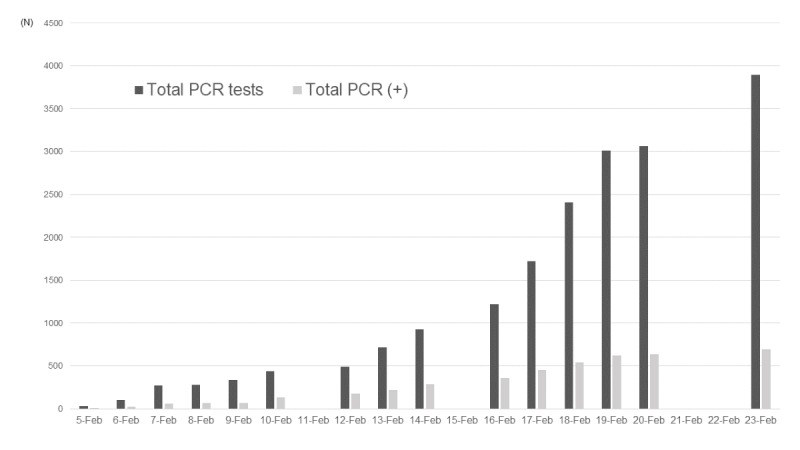
The total number of reverse transcription–polymerase chain reaction (RT-PCR) tests and positive results.

**Figure 2:**
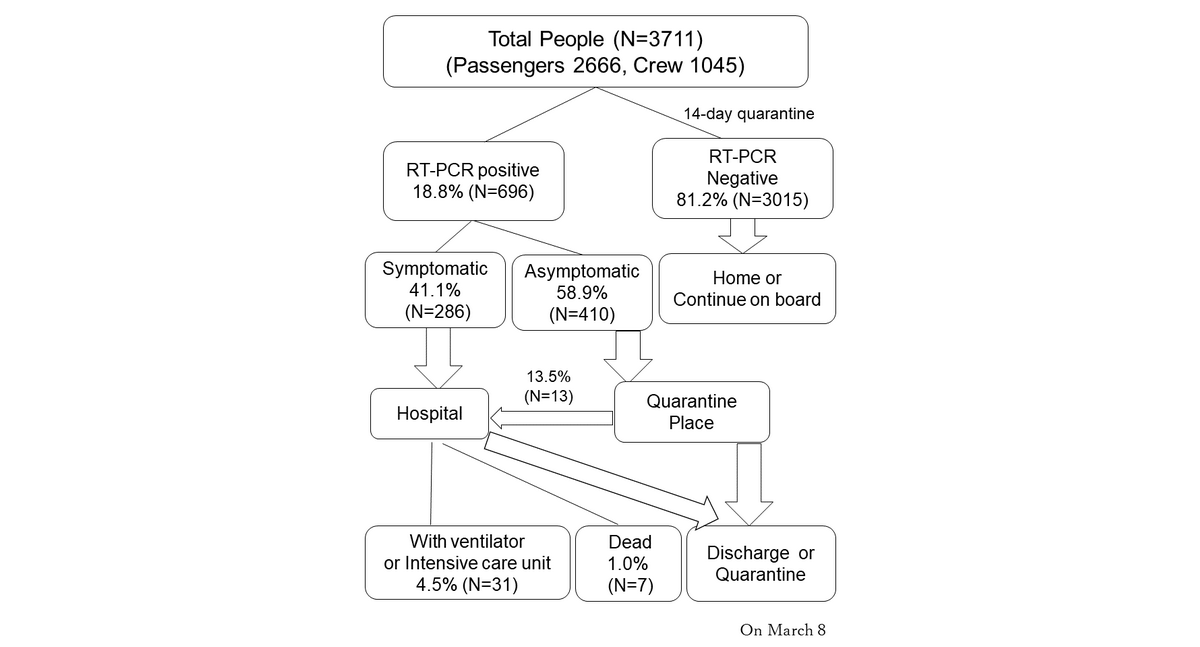
The flowchart of a large-scale cruise ship quarantine.

This report did not contain any personal information, and all information was anonymized before it was added into the report. There was no reward for the research participants, since there are no economic interests that affect the research results. This research paper was approved by the ethics committee of Yodogawa Christian Hospital (Approved No 2020-006). RT-PCR tests were performed for all 3711 passengers and crew of the Diamond Princess; 696 (18.8%) passengers tested positive, of which 410 (58.9%) were asymptomatic (Figure 1, Table 1). As of March 8, 2020, 31 patients (4.5% of those with positive RT-PCR results) were hospitalized with a ventilator or in intensive care units, and 7 patients (1.0% of those with positive RT-PCR results) had died (Figure 2) [4,5]. We transported asymptomatic people to the quarantine location. The criteria for “asymptomatic” status were no fever (body temperature <37.5 °C/99.5 °F as measured by an axillary thermometer), with an SpO_2_ (blood oxygen saturation) of 97% or above as measured with a pulse oximeter in room air. Of the 128 people who were transported, 75% (n=96) had positive RT-PCR results while on board the ship. Of the asymptomatic people with RT-PCR–positive results, 13.5% (n=13) required medical intervention after being transported from the ship and were transferred from the quarantine location to a hospital within 24 hours of arrival.

**Table 1:** Reverse transcription–polymerase chain reaction tests and the results.

Date (year 2020)	RT-PCR^a^ tests, n	Positive RT-PCR results, n (%)	Total RT-PCR tests^b^, n	Total positive RT-PCR results^c^, n (%)
5-Feb	31	10 (32.3)	31	10 (32.3)
6-Feb	71	10 (14.0)	102	20 (19.6)
7-Feb	171	41 (24.0)	273	61 (22.3)
8-Feb	6	3 (50.0)	279	64 (22.9)
9-Feb	57	6 (10.5)	336	70 (20.8)
10-Feb	103	65 (63.1)	439	135 (30.8)
11-Feb	—^d^	—	439	135 (30.8)
12-Feb	53	39 (73.6)	492	174 (35.4)
13-Feb	221	44 (19.9)	713	218 (30.6)
14-Feb	217	67 (30.9)	930	285 (30.6)
15-Feb	—	—	930	285 (30.6)
16-Feb	289	70 (24.2)	1219	355 (29.1)
17-Feb	504	99 (19.6)	1723	454 (26.3)
18-Feb	681	88 (12.9)	2404	542 (22.5)
19-Feb	607	79 (13.0)	3011	621 (20.6)
20-Feb	52	13 (25.0)	3063	634 (20.7)
21-Feb	—	—	3063	634 (20.7)
22-Feb	—	—	3063	634 (20.7)
23-Feb	831	57 (6.9)	3894	691 (17.7)

^a^RT-PCR: Reverse transcription–polymerase chain reaction.

^b^The total RT-PCR test number is larger than the number of passengers and crew on board because some people required retesting due to the appearance of new symptoms.

^c^The total number of positive RT-PCR results does not include the people who were transported by emergency disembarkation to the medical institution.

^d^Not available.

Case fatality ratios and infection fatality ratios on the Diamond Princess ship were reported to be 2.6% (95% CI 0.89-6.7) and 1.3% (95% CI 0.38-3.6), respectively [6]. Mizumoto et al [7] determined that most infections occurred before the quarantine started. However, the environment of a cruise ship is vulnerable to the spread of infection, and the peak reproduction number on the Diamond Princess ship was 12.1 before the quarantine started [8]. We will describe the medical activities and difficulties experienced in infection control on the cruise ship.

## Structure of the Cruise Ship

**Figure 3:**
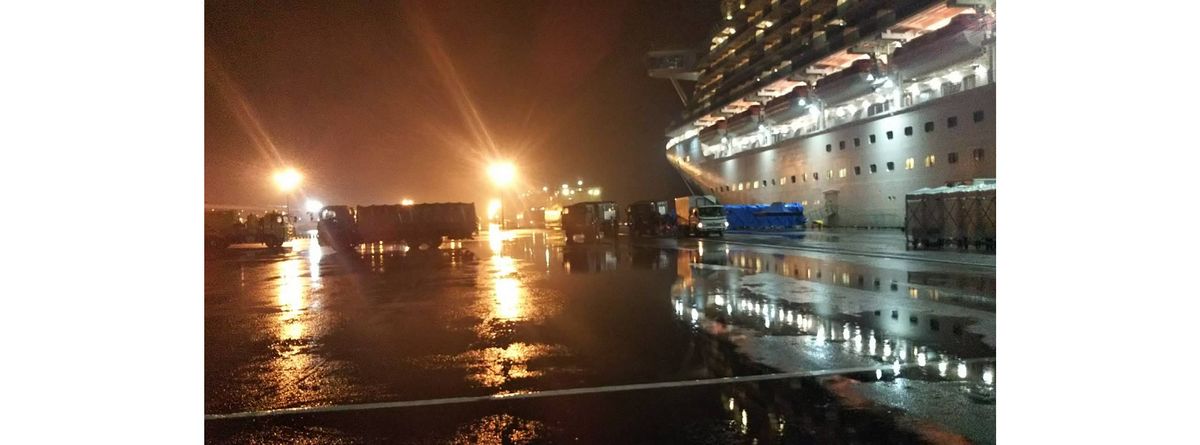
The Diamond Princess was anchored at Daikoku Wharf.

**Figure 4:**
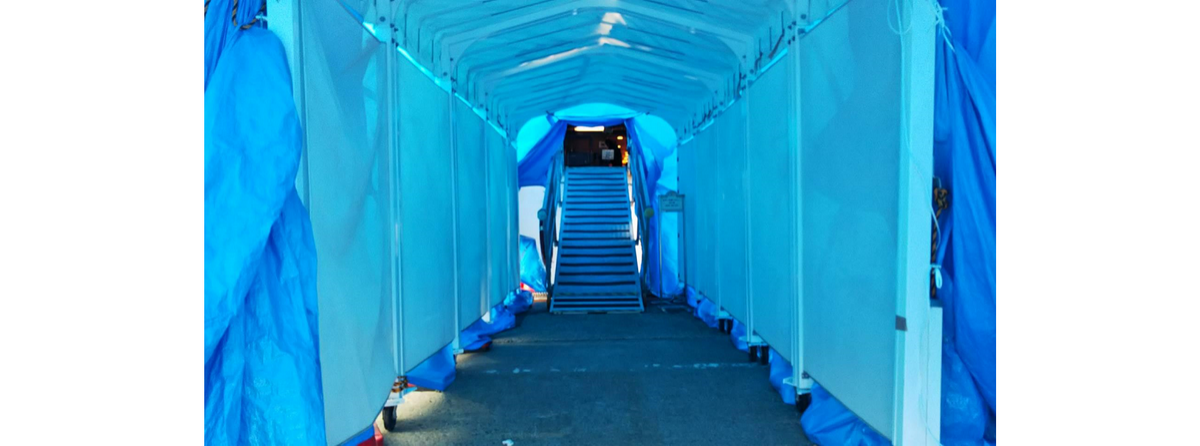
The entrance of the ship.

First, the structure of the cruise ship made it difficult to carry out the medical services required for an outbreak of an emerging infectious disease. The situation necessitated onboard quarantine with complete inspection and isolation of RT-PCR–positive persons. Additionally, today’s cruise ships are huge. The Diamond Princess had 3706 people on board, including 2706 passengers of many nationalities. The ship began service in 2004; it is 290 meters (951.4 feet) long and 37.5 meters (123.0 feet) wide, with 18 floors [9]. Each room has a toilet and shower. The high-class rooms are large and have balconies. As the class level decreases, the area of the rooms becomes smaller and the rooms are located on lower floors. The lowest class rooms are interior rooms with no windows. Crew rooms are even smaller and have limited personal space. All large cruise ships have many rooms with narrow corridors, and many people gather in small spaces, such as restaurants, theaters, and casinos. The Diamond Princess was anchored at Daikoku Wharf (Yokohama City) and berthed at the quay (Figure 3). The ship has three elevator halls: one near the bow, one near the center, and one near the stern. The opening at the stern is used only for carrying supplies, so we could only use 2 elevators to enter. From the outside of the ship, we passed through a narrow passageway and entered the ship from the opening near the center (Figure 4). Then, we passed through a security check and reached the elevator hall in front of the medical center.

## Emerging Diseases and Cruise Ships

Emerging infectious diseases are novel to humans and the mode of transmission, infectivity (basic reproduction number), severity rate, fatality rate, and long-term prognosis are not known at the time of confirmation. At the beginning of the outbreak in China, it was thought that COVID-19 could not be spread via human-to-human transmission. However, it later became clear that human-to-human infection could occur. The possibility of airborne infection was also raised. At the beginning of implementation of the quarantine, we had to enact measures based on limited information, which is confusing. In general, when we do not have the exact information, we have to consider the maximum risk possible. During the quarantine of the Diamond Princess, a lot of new information about COVID-19 in China was published; hence, we had to change our practice and attitude accordingly. There might be a gap between the new and old infection control measures.

There were several collaborators in the response to the outbreak on the Diamond Princess, such as the original medical staff; Ministry of Health, Labour and Welfare; quarantine support team; Self-Defense Forces; Disaster Medical Assistance Team (DMAT); Japanese Red Cross Society; Disaster Psychiatric Assistance Team (DPAT); Japan Medical Association Team (JMAT); and National Hospital Organization. Some staff did not have medical qualifications and each organization had different standards.

## The Quarantine Strategy of the Japanese Government

On February 3, 2020, when the ship arrived at Yokohama, a quarantine was initiated. All passengers and crew underwent medical examinations. On February 5, the RT-PCR results from the throat swab for symptomatic people and their close contacts revealed that 10 of 31 individuals were positive for SARS-CoV-2. On the same day, the Japanese government decided that all passengers were to be quarantined in their cabins for 14 days [10]. Based on international guidance on infection control, the crew continued to maintain ship functions and support passengers for their food, clothing, and shelter-related needs.

At this point, the RT-PCR testing took about 6 hours, but it took additional time to collect and transport samples and verify the results. To protect the personal information of the people involved, specimens are not managed by name but by individually identified specimen ID. Caution was required when double-checking the test results against the individual ID.

A total of 2666 passengers and 1045 crew members underwent RT-PCR testing [4,5]. We could not isolate the cabin crew since they needed to maintain the ship's functions and provide passengers with food and laundry. Of the cabin crew, those with symptoms or positive RT-PCR results disembarked and were transferred to the appropriate facilities, depending on their condition. Those with negative RT-PCR results were transferred to a residential facility to be observed for 14 days after disembarkation. We were aware that the RT-PCR test was not sensitive enough and might have led to false negatives. Those who tested positive were promptly notified that they were positive before being transferred to the hospital or quarantine facilities, under the Quarantine Law. Since a certain number of false negative RT-PCR test results were expected, the negative results were labeled as “undetermined test results” until the last day of the 14-day quarantine period and the negative result was reported at the time of the disembarkation. The RT-PCR–negative passengers who had not developed any symptoms after 14 days of cabin isolation were discharged and returned to their homes.

## Operational Difficulties

The following are 5 difficulties we faced during the quarantine of the cruise ship.

### Securing Traffic Lines

Theoretically, we needed to divide the traffic line between infectious (red zone) and noninfectious things (green zone) including humans, but exceptions were made because of the following reasons.

First, there were many elderly people over 75 years old, and some of them could not walk on their own. To separate the traffic line of the infected people from the medical staff, the other opening on the bow side was considered to be the disembarkation port for the infected people. However, it was very hard for aging passengers to walk to the entrance at the bow.

Second, going through the center entrance was the shortest way for the medical staff to get to the headquarters. The shipboard activity headquarters and the medical support headquarters were located in the two dining areas in the center of Deck 5, which is directly above the entrance in the center [9]. In addition, using that entrance meant that we did not need to use an elevator to get to the headquarters. This route minimized contact with the passengers and crew. If the bow side was used as an entrance for the medical staff, people would have had to walk through narrow corridors between cabins for a long time to reach the headquarters.

Looking back, equipped with the current information, it seems that the elevator hall in front of the medical center could have had a higher infection risk because it was not possible for infected and noninfected people to use the elevator separately. The place where the headquarters was located was where it did not overlap with other people's traffic lines.

### Coordinating Accepting Facilities and Transport Means

Under the Quarantine Act and the Infectious Diseases Act, people infected with SARS-CoV-2 were to be placed in quarantine. It was very difficult to decide where to isolate the 696 RT-PCR–positive individuals, arrange for transportation, and ensure that each person was transported to the facility. The Kanagawa Prefectural Government and its supporting DMAT were in charge of contacting the hospital to be used as the isolation facility, making inquiries about acceptance, deciding who would be placed in which facility, and securing vehicles for their transport. The DMAT command center, located at the terminal of the Daikoku Wharf, was in charge of deciding which vehicles would be used to transport the RT-PCR–positive patients. The onboard medical headquarters was in charge of checking the medical condition of the people who would disembark and of supporting them to the entrance at the center of the ship. The DMAT at the entrance of the ship was in charge of checking the preparation of the vehicles and their destination and ensuring correct transportation.

**Figure 5:**
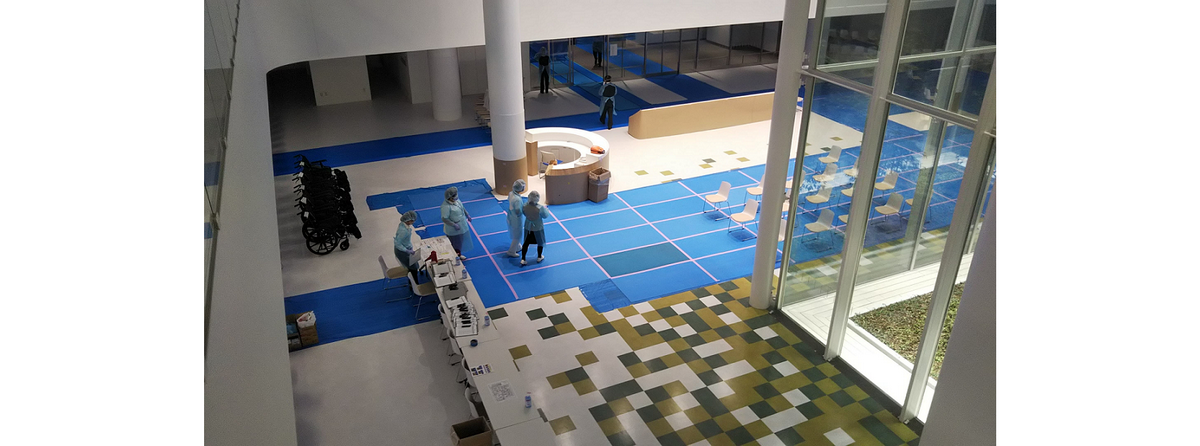
At the entrance of the quarantine building. Body temperature and SpO2 were checked while people were registered and baggage was picked up.

At first, we transported the symptomatic patients to the designated medical institutions in that area that were equipped to handle infectious diseases. However, all the beds in Kanagawa Prefecture were soon filled, and we had to extend transportation to other places in the Kanto region. At the same time, the Japanese government was operating facilities for health observation and quarantine for people who were returning to Japan on flights from Hubei Province. We had to expand the transportation area to Fukushima, Nagano, and even Osaka, which is a 6-hour drive from the ship. On the peak day, we had new 99 RT-PCR–positive patients, and we had to transport family members separately, even though they should have stayed in the same facility. We could not send all family members to the same quarantine place at that time. The Fujita Medical University Okazaki Medical Center offered to accept 170 asymptomatic patients. Since this facility was still in preparation to open as a hospital in April 2020, it was intended to only accept patients who would require no medical treatment. The author was dispatched as a DMAT for logistic support at Okazaki Medical Center and was in charge of ensuring bus transportation, room allocation, arrival confirmation, advice on the transfer to a medical facility, and overall reception. Since the patient lists were sent to the quarantine facility from Yokohama headquarters in advance, we finished the allocation of the name, ID, and room before the arrival of the bus. At the quarantine building, the transport bus was attached to the front entrance, the staff first carried their baggage to the entrance hall, and then the passengers got off the bus. We checked their body temperature and SpO_2_ levels while their baggage was picked up and patients were registered. We took face photos and attached the ID registration wristband in the allocated room (Figure 5).

### Risk of Deterioration

As previously reported, chest computed tomography (CT) has a high sensitivity (97%; 95% CI 95%-98%) for COVID-19 pneumonia [11]. We also confirmed that 54% (n=221) of the asymptomatic patients with a positive RT-PCR result had observable lung opacities on chest CTs [12]. Some people who were transported from the ship to the Okazaki facility developed mild chest pain during the 6-hour transportation. Based on the results of the CT image, the development of pneumonia may cause pleural pain. The speed at which the patients changed from being asymptomatic or mild to severe was very high. About 10% of asymptomatic people developed symptoms during a 6-hour transport, and 10%-20% of them worsened rapidly to a state in which intubation was considered within 24 hours.

### The Complex Transporting Process on the Ship

The following 6 processes were required to transfer a person from self-isolation in the cabin room to another facility:

1. Explanation to the person that his/her RT-PCR test result was positive (on board)
2. Determination of the destination (by the prefectural government)
3. Determination of the vehicle (commander in charge at the wharf)
4. Creation of the medical information report (at the onboard medical headquarters)
5. Packing of baggage (by the individuals)
6. Visiting the toilet before long-distance transportation (by the individuals)

Once the process was completed, the DMAT of the onboard medical headquarters assisted the person in moving from their cabin to the entrance of the ship. Many medical institutions preferred to accept patients in the daytime, so these tasks were concentrated in a very short time.

Unlike the usual disembarkation from a cruise ship, the patients needed to carry their baggage themselves. They had to pass through the quarantine area and customs. Some people had difficulty walking or had a lot of baggage, which meant it took a long time to move. The author was to check each transportation process and the departure of the vehicle and report it to the command center. Many foreign passengers did not understand Japanese or English at all, making the situation unimaginably difficult to manage. Sometimes, we had to ask the crew to interpret the command, even if it increased the risk of infection.

### Support for Daily Life on the Ship

We needed to support the daily lives of 3711 people on the ship. Since all passengers were isolated in each cabin, we had to deliver daily supplies to each room. There were people of various nationalities and religions aboard, and it was necessary to consider religious taboos and allergies. The crew members had to keep working under the risk of infection. The crew dining area was considered the primary area of infection for the crew, since the food service had the most confirmed cases [13]. We recommend treating the crew the same way as the passengers for infection control. In addition, a lot of water is necessary for human life and a lot of sewage was generated. In the beginning, we left the pier to the open ocean offshore once every few days for sewage disposal, but in the latter half of the quarantine period, the government and municipalities facilitated drinking water delivery and sewage water collection while the ship was still alongside the pier. Leaving the pier also became a barrier to the patients' transportation.

We have described the difficulties associated with medical activities in the management of emerging infectious diseases on a cruise ship. Infection control on a cruise ship is very difficult because of environmental factors [14], human factors, and limited medical resources.

On February 24, 2020, the World Health Organization announced an interim guidance for the operational considerations for managing COVID-19 cases and outbreaks on board ships [15]. We learned that a significant number of passengers with positive RT-PCR results had no or mild symptoms. The deterioration of patients with COVID-19 is very fast, suggesting that authorities need to prepare for patients’ transfer and the admitting hospital when disembarking the passengers. There were several difficulties on the cruise ship, such as securing traffic lines, coordinating accepting facilities and transport means, risk of deterioration, the complex transporting process on the ship, and support for daily life on the ship. We recommend treating the crew the same way as the passengers for infection control. We must make a plan for the future to protect travelers and passengers from emerging infectious diseases on cruise ships. We strongly hope this report will be helpful to the people who are working to control COVID-19 infections on cruise ships worldwide.

## Abbreviations

COVID-19: coronavirus disease
CT: computed tomography
DMAT: Disaster Medical Assistance Team
DPAT: Disaster Psychiatric Assistance Team
JMAT: Japan Medical Association Team
RT-PCR: reverse transcription–polymerase chain reaction
SARS-CoV-2: severe acute respiratory syndrome coronavirus 2
